# PHPGAT: predicting phage hosts based on multimodal heterogeneous knowledge graph with graph attention network

**DOI:** 10.1093/bib/bbaf017

**Published:** 2025-01-20

**Authors:** Fu Liu, Zhimiao Zhao, Yun Liu

**Affiliations:** College of Communication Engineering, Jilin University, No. 2699 Qianjin Street, Chaoyang District, Changchun 130012, China; School of Artificial Intelligence, Jilin University, No. 5988 Renmin Street, Nanguan District, Changchun 130022, China; College of Communication Engineering, Jilin University, No. 2699 Qianjin Street, Chaoyang District, Changchun 130012, China

**Keywords:** phage host prediction, multi-modal heterogeneous knowledge graph, graph attention network, deep learning

## Abstract

Antibiotic resistance poses a significant threat to global health, making the development of alternative strategies to combat bacterial pathogens increasingly urgent. One such promising approach is the strategic use of bacteriophages (or phages) to specifically target and eradicate antibiotic-resistant bacteria. Phages, being among the most prevalent life forms on Earth, play a critical role in maintaining ecological balance by regulating bacterial communities and driving genetic diversity. Accurate prediction of phage hosts is essential for successfully applying phage therapy. However, existing prediction models may not fully encapsulate the complex dynamics of phage–host interactions in diverse microbial environments, indicating a need for improved accuracy through more sophisticated modeling techniques. In response to this challenge, this study introduces a novel phage–host prediction model, PHPGAT, which leverages a multimodal heterogeneous knowledge graph with the advanced GATv2 (Graph Attention Network v2) framework. The model first constructs a multimodal heterogeneous knowledge graph by integrating phage–phage, host–host, and phage–host interactions to capture the intricate connections between biological entities. GATv2 is then employed to extract deep node features and learn dynamic interdependencies, generating context-aware embeddings. Finally, an inner product decoder is designed to compute the likelihood of interaction between a phage and host pair based on the embedding vectors produced by GATv2. Evaluation results using two datasets demonstrate that PHPGAT achieves precise phage host predictions and outperforms other models. PHPGAT is available at https://github.com/ZhaoZMer/PHPGAT.

## Introduction

The overuse of antibiotics has led to the emergence of bacterial-resistant bacteria, posing a significant o public health threat [1]. To combat this issue, phage therapy has emerged as a promising approach to treating bacterial infections [2]. However, the effectiveness of phage therapy hinges on accurately identifying phage hosts. Traditional experimental methods like single-cell viral tagging [3] and PCR (polymerase chain reaction) [4] are costly and have low throughput. Moreover, <1% of all phages have been successfully cultivated in laboratory settings [5], due to stringent conditions and limited bacterial host resources.

Given these challenges, there is a pressing need for more reliable and scalable methods to predict phage hosts. High-throughput sequencing technologies have revolutionized phage biology research, but they don’t directly elucidate phage–host interactions. Bioinformatics tools for predicting phage host ranges are categorized into alignment-based and alignment-free methods [6]. Despite their utility, these tools may not fully capture the intricate dynamics of phage–host interactions in complex microbial environments, suggesting room for improvement.

Viruses are among the most diverse and abundant biological entities on Earth, with an estimated 10^31^ viruses globally [7]. Within this viral domain, bacteriophages (phages), which specifically infect bacteria and archaea, constitute the majority of viral particles in nature. Phages are acknowledged as diminutive and pervasive entities in numerous ecological contexts [8]. They play a crucial role in regulating bacterial and archaeal populations and facilitating gene exchange, thus maintaining ecosystem balance on a global scale [9]. These attributes highlight their potential application in treating bacterial diseases.

Recent graph-based deep learning methods, such as HostG [10] and CHERRY [11], have demonstrated superior performance. These methods integrate information from diverse data sources and adapt dynamically as new data become available. However, due to the complexity of microbial communities, they don’t consider host–host interactions, which are essential for understanding microbial community dynamics, revealing functional redundancies among hosts, and predicting phage targeting of similar bacterial functions. Moreover, the reliance on graph convolutional networks (GCNs) in these methods can limit their capacity to capture the nuances of node relationships in heterogeneous graphs with diverse interaction types.

Similarly, heterogeneous graph methods have also been applied to drug repurposing, where they help identify new therapeutic uses for existing drugs by capturing complex interactions between drugs, targets, and diseases [12]. This success highlights the potential of graph-based methods in understanding and predicting complex biological interactions.

In light of these developments, this study introduces PHPGAT, a novel phage–host prediction model. The prominent contributions of this study are the innovative integration of multimodal data sources and advanced graph attention mechanisms, which enable PHPGAT to capture complex relationships between phages and their hosts, providing a significant advancement in phage studies. Distinguishing itself from CHERRY, which constructs its graph based on phage–phage and phage–host interactions through protein and nucleotide sequence similarity and CRISPR-Cas (Clustered Regularly Interspaced Short Palindromic Repeats–CRISPR-associated) systems [13], PHPGAT introduces a more sophisticated approach. By integrating additional features like RBPs (receptor-binding proteins) [14] and 16S rRNA (ribosomal ribonucleic acid), and by incorporating host–host interactions into the graph, PHPGAT creates a more comprehensive multimodal heterogeneous knowledge graph. This enhanced graph offers a detailed and nuanced representation of phage–host interactions, enriching our understanding of these complex relationships.

PHPGAT leverages GATv2 [15], which offers distinct advantages over traditional GCNs. GATv2 generates low-dimensional, task-specific node representations through an end-to-end training paradigm, using advanced attention mechanisms to capture and extract intricate relationships within the knowledge graph. By generating embedding vectors for each node, this process facilitates the prediction of interactions between phages and potential hosts via an inner product decoder. Evaluation results using two datasets demonstrate that PHPGAT accurately predicts phage hosts and outperforms other models, though it shows reduced performance when handling extremely long sequences and requires substantial computational resources. It is hoped that this research will provide valuable insights into the dynamics of phage–host interactions and significantly contribute to the rapidly advancing field of phage studies.

## Related work

Bioinformatics methods for predicting and estimating the hosts of phages can be broadly categorized into two primary approaches: (i) alignment-based method and (ii) alignment-free method.

(i) Alignment-based methods, which rely on shared sequences between phages and their hosts, predict host ranges based on sequence homology and similarity. Notable examples include PHIBRO [16], PHIST [17], SpacePHARER [18], and CL4PHI [19].

PHIBRO [16] utilizes the ratio of overlapping ranks derived from BLAST [20] search results to assess the similarity between phages and hosts. PHIST [17] detects shared *k*-mers to identify phage–host pairs. SpacePHARER [18] determines hosts by comparing the CRISPR [21] region with phage sequences. CL4PHI [19] employs FCGR (frequency chaos game representation) [22] to encode whole-genome sequences for zero-shot prediction. Despite their high accuracy, alignment-based models are often constrained by their dependence on existing databases.

(ii) Alignment-free methods, which rely on sequence composition and gene feature similarity, offer distinct advantages in scenarios where phage and host sequences display minimal homology or low sequence similarity. These methods categorize phage sequences by comparing their whole-genome features with a database of potential bacterial hosts (phage–host similarity) or a database of phages with known hosts (phage–phage similarity). Notable examples include VHM [23], WIsH [24], PHP [25], PredPHI [26], DeepHost [27], PHIAF [28], HostG [10], CHERRY [11], HoPhage [29], and iPHoP [30].

VHM [23] quantitatively measures the similarity between phage and host sequences by comparing nucleotide frequencies, based on the observation that phage and host genomes frequently display similar ONF (oligonucleotide frequency) patterns. The host for a given phage is predicted by identifying the host genome with the highest ONF similarity. WIsH [24] constructs an eighth-order Markov model for each potential bacterial genome, calculating the likelihood of *k*-mer matched across all conceivable phage–bacterial genome pairings.

PHP [25] employs the 4-mer frequency difference between phage and its host’s genomic DNA as the primary feature. It posits that these frequencies follow a Gaussian distribution and utilize *k*-means clustering to predict the host by assessing the likelihood ratio of the 4-mer frequency difference between an unknown phage and potential bacterial candidates.

PredPHI [26] constructs a feature vector using the proportions of amino acids, the abundance of chemical elements, and the molecular weights of proteins. It employs statistical measures, including the mean and standard deviation, as input for a deep neural network to predict the host. DeepHost [27] leverages *k*-mers across diverse sequence intervals to generate a 3D matrix that serves as input to a CNN (convolutional neural network), thereby predicting potential bacterial hosts with high fidelity. PHIAF [28] addresses the issue of sparse positive samples for phage hosts by proposing a data augmentation strategy based on generative adversarial networks (GANs). It extracts feature vectors from phage and bacterial protein sequences, performs data augmentation with GAN-generated samples, and uses a dual-layer CNN with an attention mechanism to predict phage–host compatibility.

HostG [10] constructs a semisupervised model based on GCN to predict phage hosts, incorporating nodes representing both phages and bacteria in the graph, with labeled bacteria nodes for classification. The model is trained to assign labels to phage nodes with unknown hosts. CHERRY [11], also based on GCN, predicts phage host ranges using 4-mer vectors of phage or bacterial DNA sequences as node features, employing an encoder–decoder structure and a fully connected neural network classifier.

HoPhage [29] integrates deep learning algorithms with Markov chain modeling to predict phage hosts. The HoPhage-G (Genus) module uses deep learning for genus-level pair prediction, and the HoPhage-S (Strain) module employs coding sequences from each candidate bacterial genome to construct a Markov chain model. The final host prediction is determined by averaging the outputs from both modules. iPHoP [30] combines five methods—BLAST [31], CRISPR [18], VHM [23], WIsH [24], and PHP [25]—to design a random forest model aimed at determining the optimal host for phages.

## Materials and methods

### Dataset description

**Figure 1:**
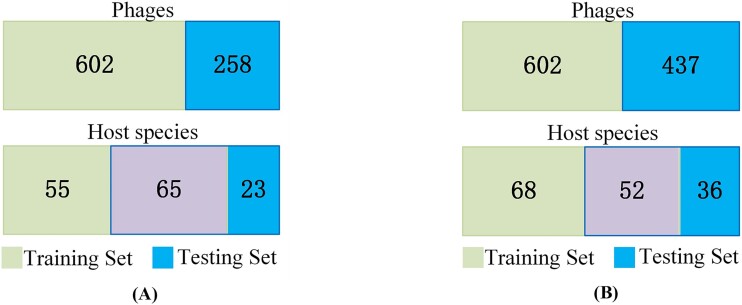
The phages and their hosts in the training and testing set (A) our dataset and (B) the CHERRY-refined dataset.

Initially, we gathered 860 phage genomes from the VHDB (Virus-Host DB) database [32], with the dataset last updated on 11 October 2023. For model development, a stratified random sampling approach was employed, designating 70% of the phage genomes for training while reserving the remaining 30% as a testing set to rigorously evaluate the model’s performance. However, due to the presence of small classes with only one or two sequences under some species labels, simple random sampling was applied to these classes to ensure adequate representation. As a result, some unique labels may be exclusively present in either the training or testing set, the detailed information is shown in Fig. 1. Our dataset encompasses 1040 documented phage–host interactions, all of which were sourced from the VHDB database and are detailed in Supplementary Data 1. Subsequently, we collected a dataset of 35 776 bacterial and 553 archaeal complete genomes from the NCBI (National Center for Biotechnology Information) RefSeq database [33] (updated on 26 October 2023), detailed information regarding this dataset is presented in Supplementary Data 2.

Additionally, to enhance the robustness and generalizability of our phage–host interaction prediction model, we supplemented the dataset partitioning method based on phage phylogenetic tree distances, as detailed in the Supplementary Material. This approach is crucial for evaluating the model’s ability to generalize to unseen, evolutionarily distant phages.

To further validate the method’s efficacy, we incorporated the testing set used in CHERRY [11] as an additional benchmark. To ensure the integrity and impartiality of the prediction outcomes, the CHERRY dataset underwent a deduplication process. During this process, any phage records with accession numbers identical to those in our dataset were excluded, thereby preserving the distinctiveness and reliability of the testing set. Following deduplication, the CHERRY-refined testing set comprises 437 interaction pairs; the detailed information is shown in Fig. 1(B). The hosts in both datasets span a wide range of taxonomic groups, as detailed in Table 1.

**Table 1:** Phage–host interactions in the two datasets.

	Our	CHERRY-refined
Phylum	15	7
Class	21	12
Order	41	25
Family	58	41
Genus	91	54
Species	143	88
Total	1040	437

### Proposed model

**Figure 2:**
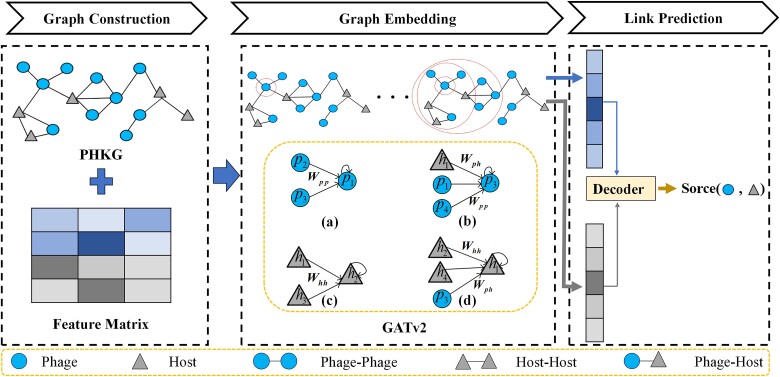
The pipeline of the proposed model.

The complete process flow of the proposed model is illustrated in Fig. 2. Initially, to enable a comprehensive analysis of diverse correlation attributes and nodal features, we construct a multimodal heterogeneous knowledge graph (PHKG) that integrates phages and their respective hosts. Furthermore, we incorporate *k*-mer frequency as a node feature to enhance the model’s learning capability.

Subsequently, we apply GATv2 to extract embedded representations from the graph, leveraging its attention mechanism to elucidate the intricate node relationships and enhance comprehension of the PHKG’s interaction dynamics.

Finally, we engage an inner product decoder to harness the learned node embeddings, thereby predicting potential phage hosts with precision. This decoder is selected for its efficiency and effectiveness in quantifying the phage–host similarity.

### Graph construction

To optimize the utilization of features derived from both training and testing samples, we construct the PHKG by establishing connections between phages and prokaryotes present in both datasets. The PHKG can be formally represented as follows:


(1) \begin{equation*} G=\left(\boldsymbol{V},\boldsymbol{E}\right) \end{equation*}

where $\boldsymbol{V}$ is the set of nodes in graph $G$, namely, node ${v}_i\in V,i=1,2,\dots, n$, including phage nodes ${p}_i\in \boldsymbol{P}$ and host nodes ${h}_i\in \boldsymbol{H}$; $\boldsymbol{E}$ is the set of edges in graph *G*, and the edge between node ${v}_i$ and ${v}_j$ can be represented as $e\left({v}_i,{v}_j\right)\in \boldsymbol{E}$.

The PHKG serves as an enhanced version that is used in the framework of CHERRY [11]. It is specifically structured, around similarities grounded in protein and nucleotide sequences, CRISPR-based similarity, 16S rRNA-based similarity, and RBP-based similarity. Depending on the nature of the connections, the graph’s edges can be categorized into three types: phage–phage, host–host, and phage–host. Each edge type of edges reflects a unique aspect of similarity or interaction between the entities it connects.

#### Phage–phage connections

To ascertain the similarity relationship between phages based on protein sequences, we employ Prodigal (v2.6.3) [34] to translate the phage genomic sequences into their corresponding protein sequences across all six open reading frames. Next, we employ DIAMOND Blastp (v2.1.9) [35] to align these proteins, retaining only alignments with an *e*-value <$1e-5$. Following alignment, the Markov Clustering Algorithm is applied to cluster the aligned proteins, generating protein clusters. Clusters that contain at least two proteins are retained, while singleton clusters are discarded. The probability of two phages sharing at least *c* clusters is:


(2) \begin{equation*} Q\left(N\ge c\right)=\sum_{i=c}^{min \left(x,y\right)}\frac{\left(\begin{array}{@{}c@{}}x\\{}i\end{array}\right)\left(\begin{array}{@{}c@{}}n-x\\{}y-i\end{array}\right)}{\left(\begin{array}{@{}l@{}}n\\{}y\end{array}\right)} \end{equation*}

where $c$ represents the count of protein clusters shared between two phages; $x$ and $y$ represent the respective total numbers of proteins found in two different phages; $N$ represents the number of common proteins; and $n$ represents the number of protein clusters.

Subsequently, to determine the similarity relationship between phages based on their nucleotide sequences, sequence alignment was conducted using BLASTN (v 2.15.0) [31]. Adhering to standard BLASTN practices, if the *e*-value resulting from the alignment between two phages is less than ${\tau}_2$, it is inferred that there exists a significant similarity between the two phage sequences.

Finally, we integrate the outcomes from both protein and nucleotide sequence–based similarity analyses to comprehensively assess relationships among phages, applying the results as:


(3) \begin{equation*} e\left({p}_i,{p}_j\right)=\left\{\begin{array}{@{}l}1\kern0.5em \mathrm{if}\ Q\left(N\ge c\right)<{\tau}_1\\{}1\kern0.5em \mathrm{if}\ BLASTN\ e- value<{\tau}_2\\{}0\kern0.5em \mathrm{otherwise}\end{array}\right. \end{equation*}

where ${\tau}_1$ and ${\tau}_2$ represent the threshold parameters indicating the existence of an edge connection in the graph. In this paper ${\tau}_1$ and ${\tau}_2$ are adopted from Shang and Sun and Johnson *et al*. [11, 31], where ${\tau}_1=1e-5$ and ${\tau}_2=1e-10$.

#### Host–host connections

For similarity based on 16S rRNA sequences, we extract sequences using Barrnap [36]. Shared 16S rRNA sequences among prokaryotes suggest a potential link between them.

Whole-genome sequence similarity is also assessed with BLASTN, with the previously used *e*-value threshold ${\tau}_2$ for significant similarity. We consolidate the outcomes from both 16S rRNA sequence similarity comparisons and whole-genome similarity analyses, applying the results as:


(4) \begin{equation*} \mathrm{e}\left({h}_i,{h}_j\right)=\left\{\begin{array}{@{}l}1\kern0.5em \mathrm{if}\ \exists\, 16\mathrm{S}\ \mathrm{rRNA}\ \mathrm{alignment}\\{}1\kern0.5em \mathrm{if}\ BLASTN\ e- value<{\tau}_2\\{}0\kern0.5em \mathrm{otherwise}\end{array}\right. \end{equation*}

#### Phage–host connections

For similarity based on CRISPR sequences, we extracted sequences using CRT [37]. Shared CRISPR sequences among phage and prokaryotes suggest a potential link between them.

For similarity based on RBP sequences, we extract sequences using PhageRBPDetection [38]. Shared RBP sequences among phage and prokaryotes suggest a potential link between them.

Whole-genome sequence similarity is also assessed with BLASTN, with the defined *e*-value threshold ${\tau}_2$ for significant similarity. We consolidate the outcomes from CRISPR sequence similarity comparisons, RBP sequence similarity comparisons, and whole-genome similarity analyses, applying the results as:


(5) \begin{equation*} \mathrm{e}\left({p}_i,{h}_j\right)=\left\{\begin{array}{@{}l}1\kern0.5em \mathrm{if}\ \exists\, \mathrm{CRISPR}\ \mathrm{alignment}\\{}1\kern0.5em \mathrm{if}\ \exists\, \mathrm{RBP}\ \mathrm{alignment}\\{}1\kern0.5em \mathrm{if}\ BLASTN\ e- value<{\tau}_2\\{}1\kern0.5em \mathrm{if}\ \exists\, \mathrm{in}\mathrm{teraction}\ \mathrm{in}\ \mathrm{dataset}\\{}0\kern0.5em \mathrm{otherwise}\end{array}\right. \end{equation*}

### Model construction

The model includes two parts, graph embedding and link prediction, as shown in Fig. 2. First, GATv2 is employed to generate the embedding representations for both the phage and prokaryote nodes within the PHKG. Then, the inner product decoder serves to generate a probability score that reflects the potential for interaction between phage ${p}_i$ and prokaryote ${h}_i$. The model employs an end-to-end training approach, dynamically fine-tuning its parameters via gradient descent.

#### Graph embedding

The input to the GATv2 is the PHKG and Feature matrix generated from the 4-mer of DNA sequences. Given the presence of both labeled nodes (phages with identified hosts) and unlabeled nodes (phages lacking host information) within the graph, the central concept of the GATv2 is to leverage the graph’s topological structure for information dissemination from labeled nodes to unlabeled ones. This process facilitates the inference of hosts for phages. The output of the GATv2 is an *n*-dimensional embedding vector that encapsulates information from phage–phage, host–host, and phage–host similarity networks derived from PHKG.

We employ GATv2 to attain efficient embeddings of node features. GATv2’s feature embedding capability is harnessed to encode the feature vectors of both phages and prokaryotes. Adjacent nodes to the focal node within the graph delineate the framework for information propagation, a construct referred to as the local computation graph of the node. All local computation graphs share parameters and weights, necessitating the application of a uniform information propagation method within each local computation graph. As shown in Fig. 2, there are four distinct local computation graphs; the node embedding is calculated as:


(6) \begin{equation*} {\boldsymbol{f}}_p={\boldsymbol{f}}_p^a+{\boldsymbol{f}}_p^b,{\boldsymbol{f}}_h={\boldsymbol{f}}_h^c+{\boldsymbol{f}}_h^d \end{equation*}

where ${\boldsymbol{f}}_p$ represents the embedding representation of phage node $P$; ${\boldsymbol{f}}_p^a$ and ${\boldsymbol{f}}_p^b$ represent the hidden states of node $P$ in the local calculation graphs (a) and (b), respectively; ${\boldsymbol{f}}_h$ represents the embedding representation of prokaryote node $H$; and ${\boldsymbol{f}}_h^c$ and ${\boldsymbol{f}}_h^d$ represent the hidden states of node $H$ in the local calculation graphs (c) and (d), respectively.

In GATv2, four local computation graphs are calculated based on the types of edges in the original graph to propagate and aggregate node information. Additionally, we integrate a multihead attention mechanism into GATv2. This method significantly enhances the exploration capability of the optimization process by diversifying the internal gradients of the model, thereby reducing the likelihood of getting trapped in local minima. After applying the multihead attention mechanism, the embedding representation of the central node is:


(7) \begin{equation*} {\boldsymbol{f}}_i^{k+1}=\delta \left(\frac{1}{M}\sum \limits_{m=1}^M\sum \limits_{\omega}\sum \limits_{j\in{\boldsymbol{N}}_{\omega}^i}{\alpha}_{ij}^{\mathrm{m}}{\boldsymbol{W}}_{\omega}^k{\boldsymbol{f}}_j^k\right) \end{equation*}

where $M$ represents the number of attention channels, and each channel uses mutually independent parameters; the model achieves optimal performance when $M$ equals 4; ${\boldsymbol{f}}_i^k\in{\mathbb{R}}^{d^k}$ represents the hidden state of node $i$ in the *k*-th layer of the GATv2, and ${d}^k$ represents the dimension of node embedding in the *k*-th layer; $\omega$ represents the type of edge in the PHKG, such as phage–phage (pp), host–host (hh), and phage–host (ph); ${\boldsymbol{W}}_{\omega}^k$ is the weight of edge type $\omega$ in the *k*-th layer, and the weight of the same type is shared; ${\boldsymbol{N}}_{\omega}^i$ represents the set of direct neighbors of the node under type, including $i$ itself; $\delta$ is the ELU activation function; and ${\alpha}_{ij}$ represents the attention weight of the composite propagation weight of the characteristics of node $i$ and the characteristics of node $j$, which is computed as:


(8) \begin{equation*} {\alpha}_{ij}=\mathrm{softmax}\left({e}_{ij}\right)=\frac{exp \left({e}_{ij}\right)}{\sum_{l\in{\boldsymbol{N}}_{\omega}^i}exp \left({e}_{il}\right)} \end{equation*}

where ${e}_{ij}$ represent the attention coefficient, which is determined by:


(9) \begin{equation*} {e}_{ij}={a}_{\omega}^k\sigma \left({\boldsymbol{W}}_{\omega}^k\left[{\boldsymbol{f}}_i^k,{\boldsymbol{f}}_j^k\right]\right) \end{equation*}

where ${a}_{\omega}^k\left(\cdot \right)$ represents a feedforward neural network in GATv2, used to transform high-dimensional data into a single real number representation; $\sigma$ represents the LeakyRelu activation function.

#### Link prediction

The inner product decoder is utilized to process the embedding vectors produced by the GATv2. This decoder intends to ascertain the likelihood of interaction between phage ${p}_i$ and prokaryote ${h}_i$, which is computed as:


(10) \begin{equation*} {Q}_{ph}= decoder\left({\boldsymbol{f}}_p,{\boldsymbol{f}}_h\right)={\boldsymbol{f}}_p^T\cdot{\boldsymbol{f}}_h \end{equation*}

where ${\boldsymbol{f}}_p$ and ${\boldsymbol{f}}_h$ are the embedding vectors of phage $p$ and host $h$.

### Loss function and model training

To optimize the phage recognition task, the model employs the Bayesian Personalized Ranking (BPR) loss function coupled with negative sampling, which is defined as:


(11) \begin{equation*} Loss=-\sum \limits_{p=1}^U\sum \limits_{h\in{\boldsymbol{N}}_p^{p2h}}\ln \left[\mathrm{sigmod}\left({\hat{Q}}_{ph}-{\hat{Q}}_{ps}\right)\right] \end{equation*}

where ${\hat{Q}}_{ps}={\boldsymbol{f}}_p^T\cdot{\boldsymbol{f}}_{\mathrm{s}}$, $s$ represents the prokaryotic node obtained through negative sampling that has no connection with phage $p$; $U$ represents the total number of phages; and ${\boldsymbol{N}}_p^{p2h}$ represents the set of prokaryotic nodes that have an edge relationship with phage $p$.

The BPR loss function strives to guarantee that the predicted probability scores for observed phage–host interactions surpass those for unobserved phage–host interactions. This approach bolsters the model’s discriminative capacity and elevates its overall performance.

To refine the model and enable a more exhaustive grasp of the interaction dynamics between phage and host, the Adam optimization algorithm [39] was employed for the training phase.

## Results and discussions

### Evaluation indicators

In this paper, accuracy and $hit@k$ are utilized to evaluate the prediction performance. Accuracy is the proportion of the number of correctly predicted samples and the total number of samples.

$hit@k$ evaluates whether the top *k* predictions of a model contain the real target of phage and is defined as:


(12) \begin{equation*} hit@k=\frac{\sum_{i=1}^N\gamma }{N} \end{equation*}

where $\gamma$ is determined by:


(13) \begin{equation*} \gamma =\left\{\begin{array}{@{}l}1\kern0.5em \mathrm{if}\ {\boldsymbol{S}}_k\cap \boldsymbol{K}\ne \varnothing \\{}0\ \mathrm{otherwise}\end{array}\right. \end{equation*}

where ${\boldsymbol{S}}_k$ is the set of the top *k* predictions; $\boldsymbol{K}$ represents the set of real hosts of a phage; and $N$ represents the total number of samples.

Moreover, since PHKG encompasses all plausible hosts and the prediction count matches the tally of scrutinized phages, this circumstance renders recall and precision equivalent to accuracy.

### Contrast experiment

To evaluate the effectiveness of the proposed model, we selected four prominent phage host recognition methods: CHERRY [11], which uses 4-mer vectors and the GCN; CL4PHI [19], which employs FCGR for zero-shot prediction; DeepHost [27], which leverages 3D *k*-mer features and the CNN; and iPHoP [30], which integrates BLAST, CRISPR, VHM, WIsH, and PHP into a random forest model. These methods represent the state of the art in phage host recognition. To ensure the accuracy of the experiment, the results of other models were updated to the latest NCBI standards; the particulars of these amendments are shown in Supplementary Data 3. In the comparative experiments, we utilized the pretrained models with default parameters as provided by the respective authors.

### Evaluation on two testing sets

#### Results of pretrained models

**Figure 3:**
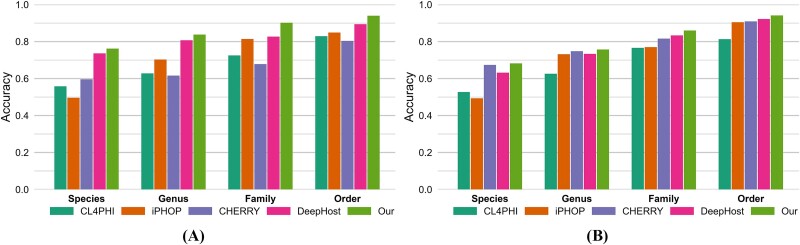
Accuracy comparison across different taxonomic levels of different models on (A) our and (B) CHERRY-refined testing sets.

Table. 2 and Fig. 3 present the accuracy values across different taxonomic levels for the two testing sets. In our testing set, DeepHost outperforms the other three comparative models across all four taxonomic levels. However, compared to DeepHost, the proposed model further improves accuracy by 4.6%, 7.5%, 3.1%, and 3.4% at the Order, Family, Genus, and Species levels, respectively. In the CHERRY-refined testing set, DeepHost surpasses CHERRY at the Order and Family levels but not at the Genus and Species levels. Nevertheless, the proposed model enhances accuracy across all four levels by 1.9%, 2.7%, 0.9%, and 0.8%, respectively.

**Table 2:** Prediction accuracy on the two testing sets.

Method	Our testing set				CHERRY-refined testing set			
	Order	Family	Genus	Species	Order	Family	Genus	Species
CHERRY	0.804	0.678	0.616	0.596	0.909	0.816	0.748	0.674
DeepHost	0.894	0.827	0.807	0.736	0.922	0.833	0.733	0.632
iPHoP	0.849	0.814	0.703	0.496	0.905	0.770	0.732	0.493
CL4PHI	0.829	0.725	0.628	0.558	0.813	0.766	0.626	0.527
Our	**0.940**	**0.902**	**0.838**	**0.762**	**0.941**	**0.860**	**0.757**	**0.682**

The best and second-best results are written in bold and underlined format, respectively.

**Figure 4:**
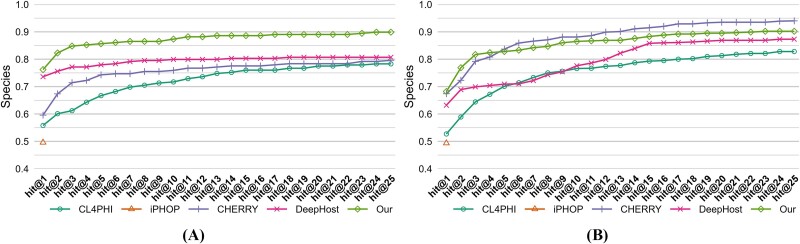
*Hit@k* comparison at the species level of different models on (A) our and (B) CHERRY-refined testing sets.

To further validate the efficacy of our method in multihost prediction scenarios, we conducted a detailed evaluation of its predictive capabilities, ranging from hit@1 to hit@25, specifically at the species level for the two testing sets, as depicted in Fig. 4. Due to the unique configuration of iPHoP, our analysis was limited to hit@1, excluding other metrics. In our testing set, the proposed model demonstrates superior performance across the entire spectrum of hit rates from hit@1 to hit@25. In the CHERRY-refined testing set, while the proposed model excels at hit rates from hit@1 to hit@4, a decline in performance is observed from hit@5 to hit@25, where its hit rates fall below those of the CHERRY model.

We have conducted an analysis of the data points contributing to the lower hit@ > 5 metrics in Fig. 4B for our proposed approach compared to CHERRY. We found that the majority of these phages are unseen by our model during training but seen by CHERRY. This suggests that the difference in performance might be attributed to the novelty of the data our model encounters during testing. Since our model has not seen similar sequences during training, it performs less effectively when predicting such novel data points. Conversely, CHERRY’s exposure to more similar sequences during training may explain its better performance in these specific cases.

#### Results of retrained model

**Figure 5:**
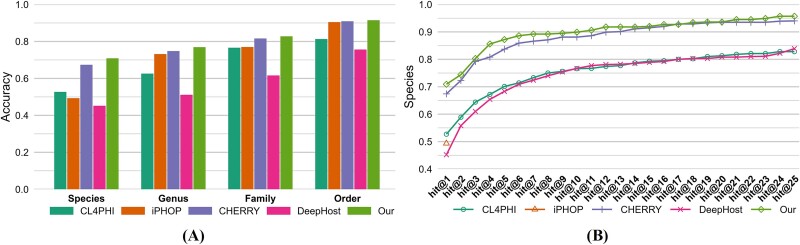
The comparative experimental results. (A) Accuracy comparison across different taxonomic levels of different models, and (B) *hit@k* comparison at the species level of different models.

Given this insight into the impact of data novelty on model performance, we sought to validate our model’s performance and ensure a fair comparison by retraining all models using the CHERRY training set. Since iPHoP does not support retraining, we utilized its pretrained version. We then evaluated all models on the CHERRY-refined testing set to assess their performance consistently. Table 3 and Fig. 5 present the accuracy values across different taxonomic levels; CHERRY outperforms the other three comparative models across all four taxonomic levels. Notably, CHERRY’s performance remains consistent between Table 2 and Table 3 because it uses the same training set as before. However, compared to CHERRY, the proposed model further improves accuracy by 0.6%, 1.2%, 2.1%, and 3.5% at the Order, Family, Genus, and Species levels, respectively.

**Table 3:** Prediction accuracy on the CHERRY-refined testing set.

Method	Order	Family	Genus	Species
CHERRY	0.909	0.816	0.748	0.674
DeepHost	0.756	0.616	0.511	0.452
iPHoP	0.905	0.770	0.732	0.493
CL4PHI	0.813	0.766	0.626	0.527
Our	**0.915**	**0.828**	**0.769**	**0.709**

The best and second-best results are written in bold and underlined format, respectively.

To further validate the efficacy of our method in multihost prediction scenarios, we conducted a detailed evaluation of its predictive capabilities, ranging from hit@1 to hit@25, specifically at the species level, as depicted in Fig. 5B, the proposed model demonstrates superior performance across the entire spectrum of hit rates from hit@1 to hit@25.

### Performance on different phage lengths

**Figure 6:**
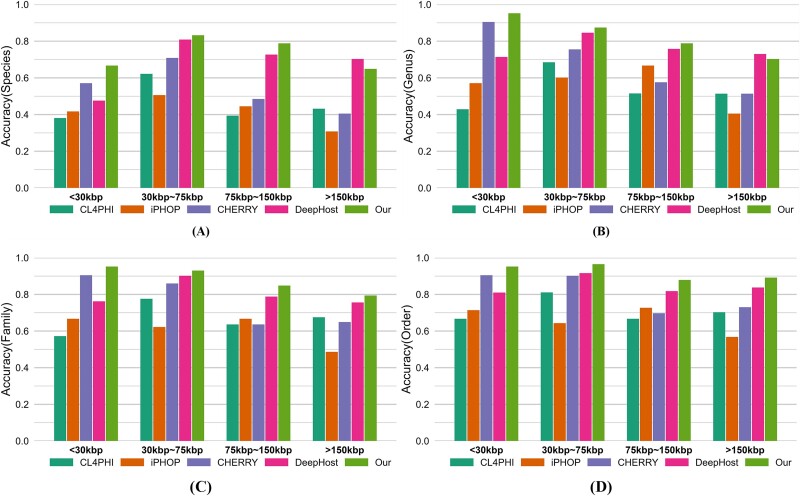
Accuracy comparisons of different models on different phage lengths at (A) species (B) genus (C) family, and (D) order levels.

Although our method achieves leading performance in prediction accuracy, the accuracy rate for species-level classification remains below 0.8. To explore the factors contributing to this limitation, we categorized phage genomes according to their natural distribution into four length intervals: “<30 kbp,” “30–75 kbp,” “75–150 kbp,” and “>150 kbp” on our testing set. As illustrated in Fig. 6, our model performs optimally across all length intervals at the Order and Family levels. However, within the “>150 kbp” interval at the Species and Genus levels, DeepHost demonstrates superior performance.

Our analysis indicates that there are two main factors contributing to this phenomenon: first, the proposed architecture, while effective for a wide range of genome lengths, faces challenges when dealing with the increased complexity and computational demands associated with longer genomes. Longer sequences can introduce additional noise and require more sophisticated mechanisms to capture long-range dependencies, which may not be fully addressed by the current model design. Second, the representation of long genomes within the training dataset is limited; in our current dataset, genomes longer than 150 kbp account for <10%, which means the model has limited exposure to such cases. This lack of diversity in the training data limits the model’s ability to generalize well to longer genomes during testing.

### Performance on different phage families

**Figure 7:**
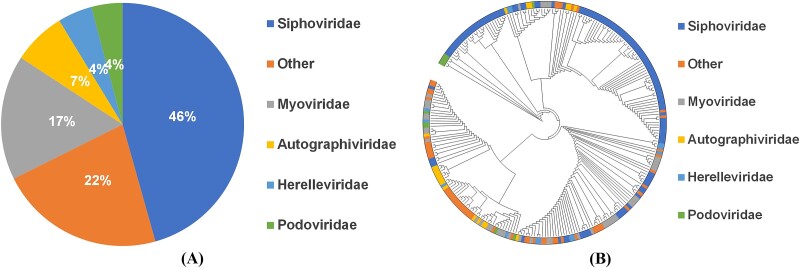
The distribution of different phage families is shown in (A) the classification of phages and (B) the phylogenetic tree.

To elucidate the relationship between model efficacy and phage family classifications, a targeted comparative study was conducted on our testing set. The classification of phages is depicted in Fig. 7A, and the phylogenetic tree is depicted in Fig. 7B. For clarity and analytical focus, categories with fewer than 10 samples were consolidated under the “Other” classification.

**Figure 8:**
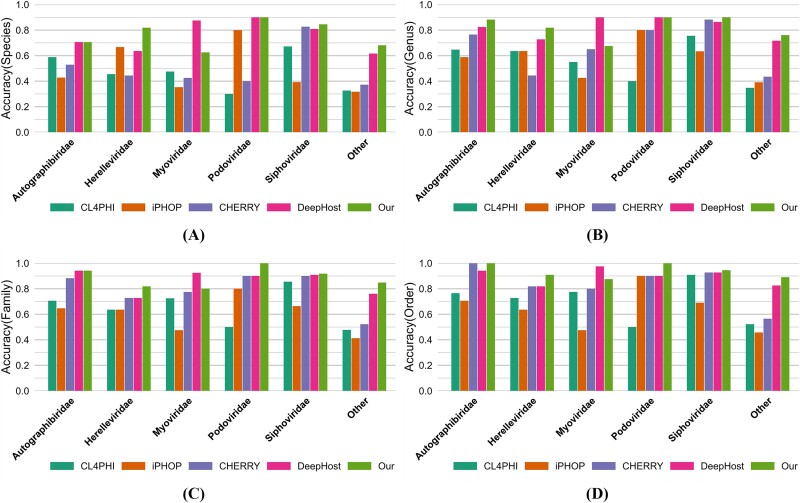
Accuracy comparisons of different models on different phage families at (A) species, (B) genus, (C) family, and (D) order levels.

As shown in Fig. 8, the results indicate that, except for a slightly lower accuracy in the “Myoviridae” family compared to DeepHost, the proposed model outperforms the other five categories. A primary reason for this discrepancy is that the majority of phages in the “Myoviridae” family have genomes exceeding 150 kbp in length.

Furthermore, comparative evaluations on the testing set based on phage phylogenetic tree distances, experiments involving unseen phages, utilization of computational resources, and statistical tests are detailed in Supplementary Material.

### Ablation study

To evaluate how individual components contribute to the prediction performance, we constructed ablation experiments by eliminating edge types and particular modal features from PHKG. Furthermore, we eliminated the model’s attention mechanism to assess its impact on overall performance.

**Figure 9:**
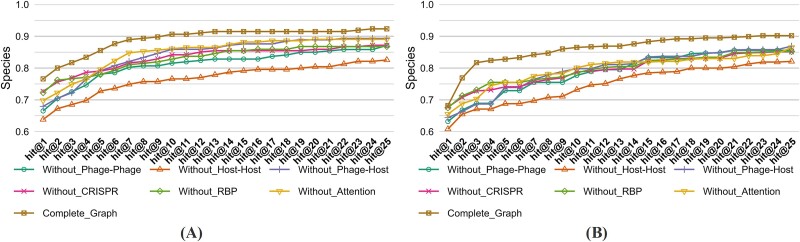
*Hit@k* with different ablation combinations on (A) our and (B) CHERRY-refined testing sets.

Table 4 demonstrates the accuracy of different ablation combinations on two testing sets at the species level. Furthermore, Fig. 9 presents the hit@k values for the same two datasets. Notably, the complete model achieves the highest performance across all hit@k metrics, ranging from hit@1 to hit@25. These findings highlight that integrating phage–phage, host–host, phage–host, CRISPR features, RBP attributes, and the model’s attention mechanism significantly enhances the model’s generalizability and predictive accuracy.

**Table 4:** Host prediction accuracy with different ablation combinations on the two testing sets.

Method	Our testing set	CHERRY-refined testing set
Without phage–phage	0.665	0.632
Without host–host	0.638	0.608
Without phage–host	0.679	0.644
Without CRISPR	0.726	0.677
Without RBP	0.722	0.675
Without attention	0.698	0.655
Complete graph	**0.762**	**0.682**

The best results are written in bold, respectively.

### Case study

#### Case 1

“*Klebsiella pneumoniae*” is a prevalent Gram-negative bacterium, notable for its pathogenicity, particularly as a causative agent of pneumonia. This bacterium has emerged as a significant health threat due to its ability to cause infections, exacerbated in recent years by the rise of drug-resistant strains, presenting a formidable challenge to clinical management.

This section analyzes the nodes in the PHKG related to “*K. pneumoniae*.” As shown in Table 5, there are six test nodes in the node adjacent to “*K. pneumoniae*”; our model predicted more correct hosts compared to other models.

**Table 5:** Host predictions for six cases.

Phage accession	Real host species	Our	CHERRY	DeepHost	iPHoP	CL4PHI
NC_021772	*Salmonella enterica*	√	√	√	×	√
NC_049818	*Escherichia coli*	√	×	×	√	×
NC_049823	*E. coli*	√	√	√	√	×
NC_049444	*Klebsiella pneumoniae*	×	×	×	×	×
NC_047850	*K. pneumoniae*	√	√	√	×	√
NC_049846	*K. pneumoniae*	√	√	√	×	√

“√” or “×” represents whether the method predicts correctly or not.

**Table 6:** Host range on species level and the host for phages.

Phage		Host species
Accession	Strain	
NC_052986	Aeromonas phage BUCT551	*Aeromonas hydrophila*
		*A. veronii*
NC_048674	Aeromonas phage AS-sw	*A. hydrophila*
		*A. salmonicida*
NC_048001	Salmonella phage SP3	*Salmonella enterica*
		*Escherichia coli*

**Table 7:** Top five host predictions of NC_052986 of different methods.

Rank	CL4PHI	iPHoP	DeepHost	CHERRY	Our
1	*Aeromonas salmonicida*	***A**.** hydrophila***	Pseudomonas fluoresce-ens	*Cronobacter sakazakii*	***A. hydrophila***
2	*Mycobacterium tuberculosis*	*Desertimonas flava*	*Klebsiella pneumoniae*	*Salmonella enterica*	*A. rivipollensis*
3	*Propionibacterium acnes*	*Phytohabitans rumicis*	*Streptomyces scabiei*	*Burkholderia pseudomallei*	*Xylella fastidiosa*
4	*K. pneumoniae*	*Alcanivorax hongdengensis*	*Edwardsiella tarda*	*Escherichia coli*	*Pseudomonas fluorescens*
5	*Propionibacterium freudenreichii*	*Aquisalimonas asiatica*	*Rhodococcus rythropolis*	*Burkholderia thailandensis*	*A**.** veronii*

Bold text indicates a species with accurate predictions.

**Table 8:** Top five host predictions of NC_048674 of different methods.

Rank	CL4PHI	iPHoP	DeepHost	CHERRY	Our
1	*Vibrio harveyi*	*Aeromonas schubertii*	***A**.** hydrophila***	*A. salmonicida*	***A. salmonicida***
2	*Pseudomonas putida*	*A. popoffii*	*Vibrio cholerae*	*Escherichia coli*	***A. hydrophila***
3	*V. alginolyticus*	***A. salmonicida***	*E. coli*	*Klebsiella pneumoniae*	*A. veronii*
4	*Bacillus cereus*	*A. piscicola*	***A. salmonicida***	*Serratia marcescens*	*A. rivipollensis*
5	*Propionibacterium acnes*	*Aeromonas bestiarum*	*Thermus thermophilus*	*Salmonella enterica*	*K. pneumoniae*

Bold text indicates a species with accurate predictions.

**Table 9:** Top five host predictions of NC_048001 of different methods.

Rank	CL4PHI	iPHoP	DeepHost	CHERRY	Our
1	***Escherichia coli***	*Sphingobacterium kitahiroshimense*	***Salmonella enterica***	***E. coli***	***S. enterica***
2	*Citrobacter freundii*	*Ornithinibacillus globulus*	***E. coli***	*Vibrio cholerae*	***E. coli***
3	*V. cholerae*	*Saccharicrinis marinus*	*Shigella sonnei*	***S. enterica***	*Klebsiella pneumoniae*
4	*Yersinia enterocolitica*	*Pseudofrancisella aestuarii*	*Paenibacillus larvae*	*Vibrio alginolyticus*	*Shigella flexneri*
5	*V. parahaemolyticus*	***E. coli***	*Y. enterocolitica*	*V. parahaemolyticus*	*S. sonnei*

Bold text indicates a species with accurate predictions.

#### Case 2

To further scrutinize the precision of our model in forecasting multiple hosts, there is a selection of three phage types from the testing set, as delineated in Table 6. These phages exhibit a noteworthy inhibitory impact on bacteria implicated in foodborne illnesses. Employing the method to forecast the top five potential hosts, as shown in Tables 7–9, the research results show that our method has successfully predicted all the hosts of these three phages, while other methods have not achieved this effect. Consequently, our method’s performance in predicting multiple hosts markedly surpasses that of the four alternative methods assessed.

## Conclusion

Motivated by the growing concern over antibiotic resistance and the urgent need for alternative therapeutic strategies, this study introduces a novel model for phage host recognition by framing the task as a multimodal knowledge graph link prediction problem. Antibiotic-resistant bacteria pose a significant threat to global health, necessitating the development of more accurate and reliable methods for identifying potential phage–host interactions. Accurate prediction of phage hosts is essential for the successful application of phage therapy, which offers a promising solution to combat multidrug-resistant pathogens.

Our approach integrates diverse data sources, including protein and nucleotide sequence similarities among phages, 16S rRNA information and sequence similarities among prokaryotes, sequence homology between phages and prokaryotes, CRISPR information, and RBP data. We employ GATv2 to generate feature embeddings and utilize an inner product decoder to estimate the likelihood of interactions between phages and prokaryotes. Experimental results demonstrate that our model surpasses existing approaches across all evaluated metrics.

Despite these significant advancements, opportunities remain for optimization in phage host prediction. Key areas for improvement include handling sequences of extreme lengths and addressing the class imbalance. Future work should aim to enrich the feature set beyond sequence-based characteristics, incorporating additional attributes such as protein tertiary structures. Developing more efficient and rapid methods for graph construction to minimize computational resource use will be essential. Leveraging large-scale pretrained methods and adopting ensemble learning methodologies could also improve model performance. Furthermore, addressing dataset imbalances through advanced data augmentation techniques and enhancing strain-level recognition capabilities will be crucial to meeting the stringent precision requirements of clinical applications.

**Key Points**

- In this work, we present PHPGAT, a new tool for predicting phage hosts based on a multimodal heterogeneous knowledge graph with GATv2 (graph attention network v2).
- To capture the intricate relationships between biological entities, we construct a multimodal heterogeneous knowledge graph by integrating phage–phage, host–host, and phage–host interactions. This graph integrates diverse types of information, including protein and nucleotide sequence similarity, CRISPR-based similarity, 16S rRNA-based similarity, and RBP-based similarity.
- Our rigorous test of PHPGAT on other datasets and the benchmark experiments against four recently published tools show that PHPGAT is the most accurate host prediction tool.

## Supplementary Material

Supplementary_Data_1_Phage-Host_interation_information_bbaf017

Supplementary_Data_2_Host_information_bbaf017

Supplementary_Data_3_Update_hostTaxonomy_information_bbaf017

Supplementary_Material_bbaf017

## Data Availability

All data and codes used for this study are available online or upon request to the authors. The source code of PHPGAT is available via: https://github.com/ZhaoZMer/PHPGAT.
