# Supplementary material for: PHPGAT: predicting phage hosts based on multimodal heterogeneous knowledge graph with graph attention network
Source: Brief Bioinform. 2025 Jan 20;26(1):bbaf017. doi: 10.1093/bib/bbaf017 (PMC11745545; doi:10.1093/bib/bbaf017)
Supplement: Supplementary_Material_bbaf017 [file supplementary_material_bbaf017.docx]

**Supplementary material**

**Phylogenetic-Based Partitioning of the Data Set**

We conducted sequence alignment on the 860 sequences within our dataset using Clustal Omega [1] to generate a distance matrix. This matrix was subsequently used to visualize the phylogenetic tree, as depicted in Fig. S1. Several branches were selected as the test set, denoted by the color-coded branches within the tree. These branches collectively represent a total of 244 phage sequences.


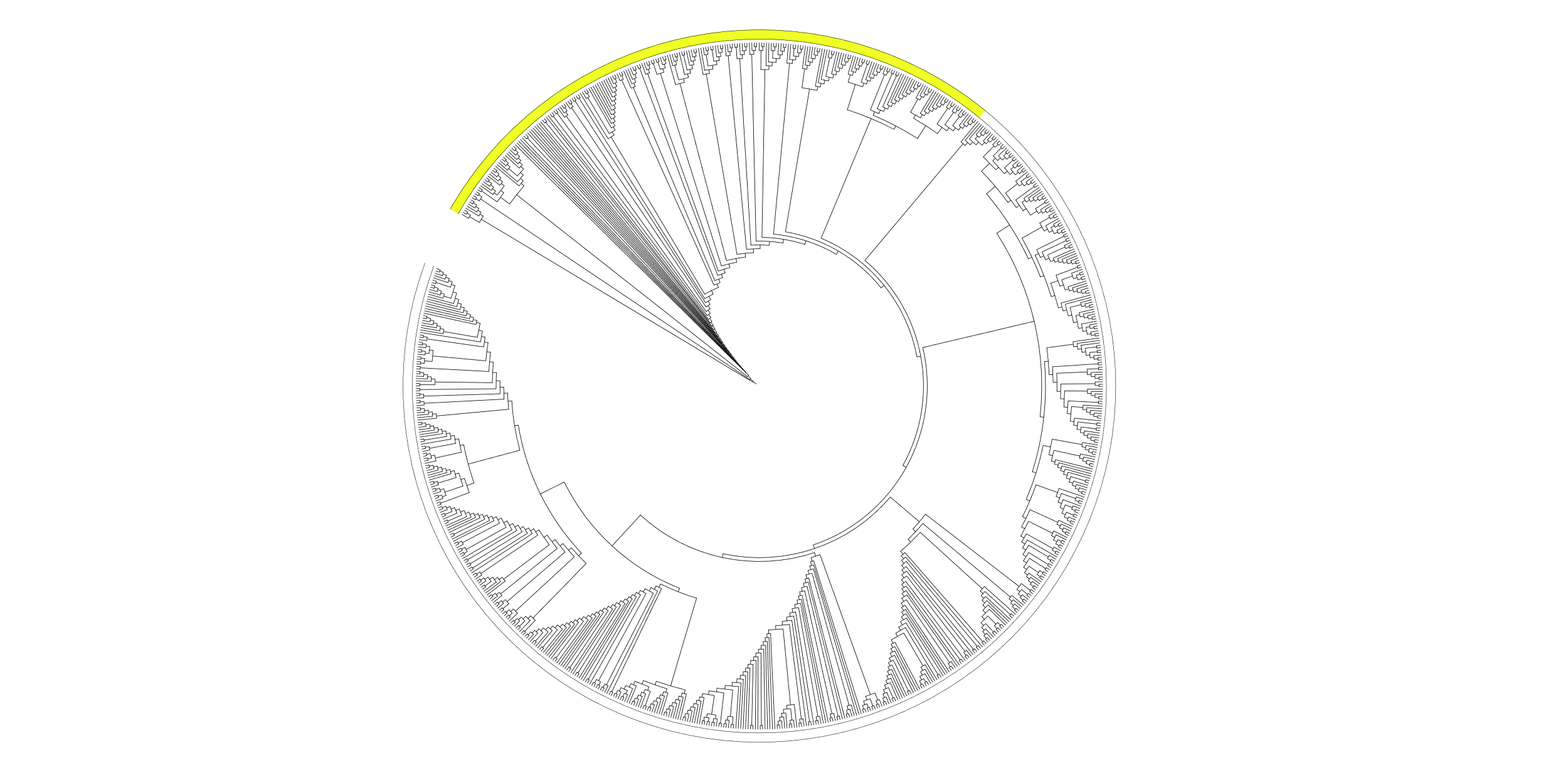


**Figure S1.** Phage phylogenetic tree based on our dataset. The phages marked with color coding represent the testing set.

**Comparative evaluation on testing set according to phage phylogenetic tree distances**

In this comparative experiment, we chose to retrain the models for consistency and fairness. However, given that iPHoP does not support retraining, a pre-trained model of iPHoP was utilized instead. We used the training and test sets as depicted in Fig. S1 for training and testing the models.

Table S1 and Fig. S2 (A) present the accuracy values across different taxonomic levels, CHERRY outperforms the other three comparative models across all four taxonomic levels. However, compared to CHERRY, the proposed model further improves accuracy by 7.6%, 6.2%, 4.4%, and 5.3% at the Order, Family, Genus, and Species levels, respectively.

To further validate the efficacy of our method in multi-host prediction scenarios, we conducted a detailed evaluation of its predictive capabilities, ranging from hit@1 to hit@25, specifically at the species level, as depicted in Fig. S2 (B), the proposed model demonstrates superior performance across the entire spectrum of hit rates from hit@1 to hit@25.

Table S1. Prediction accuracy on the testing set. The best and second-best results are written in bold and underlined format respectively.

| Method | Order | Family | | Genus | | Species | | |  |
| --- | --- | --- | --- | --- | --- | --- | --- | --- | --- |
| CHERRY | 0.851 | | 0.806 | | 0.747 | | 0.668 | |  |
| DeepHost | 0.775 | | 0.671 | | 0.556 | | 0.471 | |  |
| iPHoP | 0.833 | | 0.798 | | 0.632 | | 0.478 | |  |
| CL4PHI | 0.784 | | 0.632 | | 0.599 | | 0.418 | |  |
| OUR | **0.927** | | **0.864** | | **0.791** | | **0.721** | |  |
| **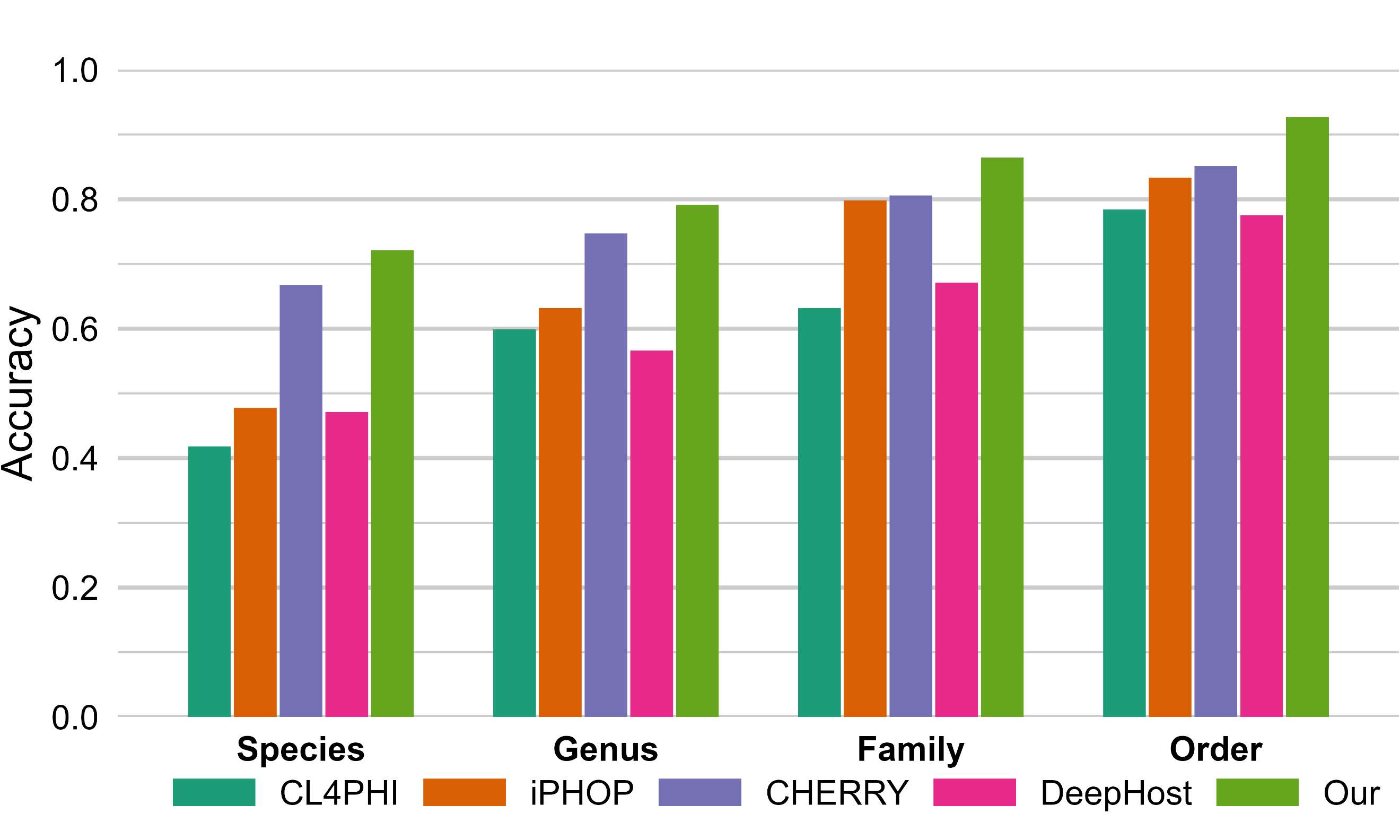**  **(A)** | | | | | | | | **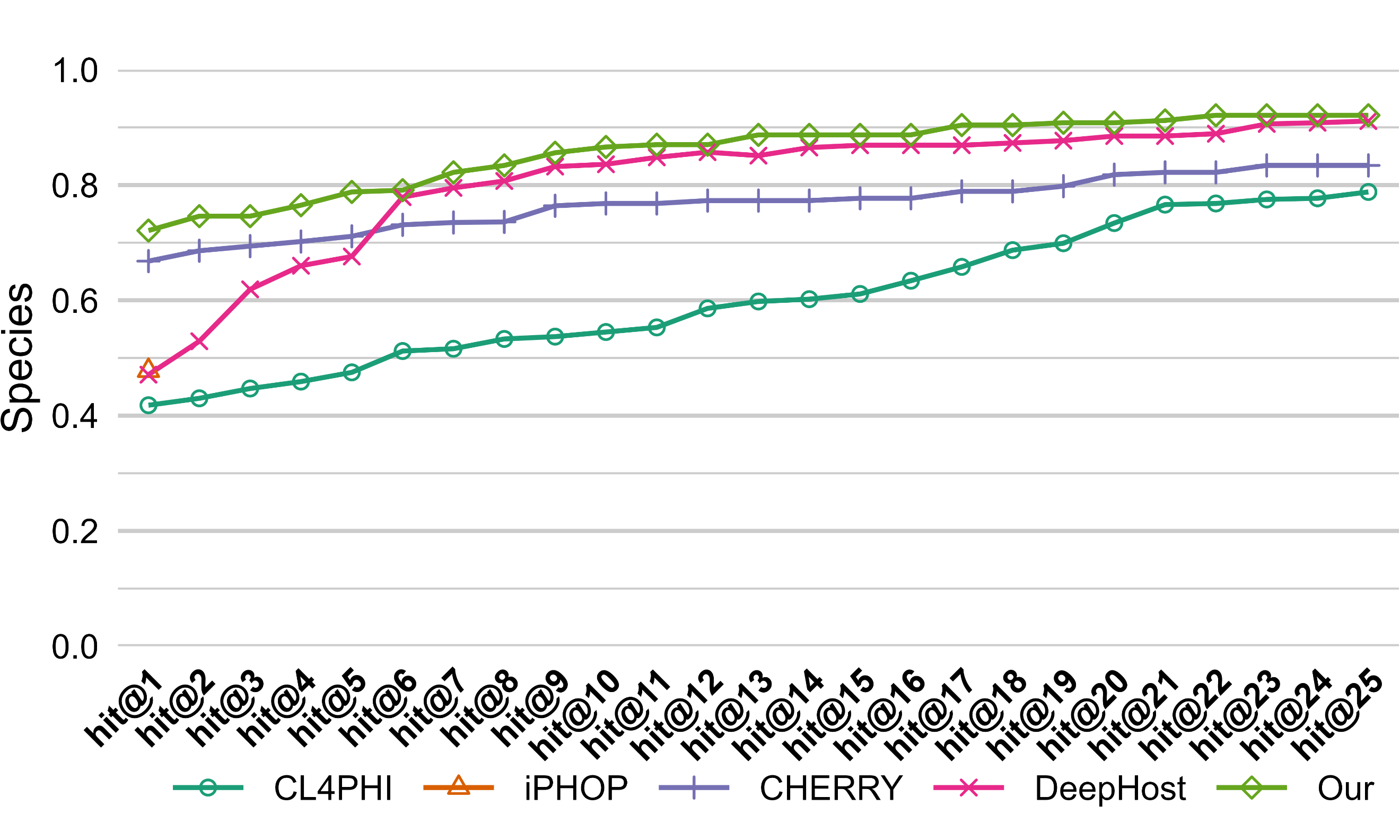**  **(B)** | |

**Figure S2.** The comparative experimental results. (A) Accuracy comparison across different taxonomic levels of different models, and (B) *hit@k* comparison at the species level of different models.

**PHPGAT can be generalized to unseen host species**

Due to limitations in model configurations, DeepHost and iPHoP can only predict phages with pre-defined labels; therefore, in this part, our method is compared only with CHERRY and CL4PHI. After removing label redundancies between the sequences in the test sets shown in Figure 1 and the training sets used by the three models, we obtained a total of 43 sequences representing 26 independent taxa. The results of these 43 sequences are compared, as shown in Table S2, and the findings indicate that our model achieves the best performance.

Table S2. Prediction accuracy on the 43 unseen phages at the species level. The best is written in bold.

| Method | Our | CL4PHI | CHERRY |
| --- | --- | --- | --- |
| ACC | **0.372** | 0.256 | 0.326 |

**Computational requirements for PHPGAT**

Our experiments were conducted on a workstation configured with an Intel(R) Xeon(R) Gold 5220 CPU and an NVIDIA A40 GPU. The time usage for each phase, peak RAM usage, and peak GPU memory usage, as shown in Table S3, indicate that the model testing time includes the duration required to construct the supplementary graph for the testing set. Notably, the model training needs to be performed only once; for future applications, it will be sufficient to add new test nodes and edges to the existing graph structure. Additionally, our model supports running solely on the CPU.

Table S3. The time usage for each phase.

| Phase | Graph Construction | Model Training | Model Testing (per sequence) |
| --- | --- | --- | --- |
| Time | 32 h 27 min | 4 h 8 min | 42s |
| Peak RAM usage | 1.51G | 3G | 1G |
| Peak GPU memory usage | 0 | 16.44G | 25.42M |

To mitigate the computational cost and complexity associated with our method, we propose several strategies for future development. These include improving graph construction algorithms to use techniques such as sparse matrix representations and incremental updates, which can minimize the resources required for maintaining and processing the graph structure. Additionally, optimizing the graph attention network (GATv2) implementation could lead to faster convergence and reduced training times through pruning redundant connections, employing mixed-precision training, and utilizing hardware-specific optimizations. Implementing feature selection techniques to identify the most informative features can reduce dimensionality and simplify the model without compromising predictive power. Utilizing batch processing techniques can improve efficiency by parallelizing computations across multiple samples or subgraphs. Advanced data augmentation techniques can help manage dataset imbalances without increasing the computational burden excessively by generating synthetic data that is computationally less intensive than retraining on larger datasets. By implementing these strategies, we aim to reduce the computational demands of our method, thereby facilitating its practical application and wider adoption.

**Statistical tests**

The Friedman test method was used to perform a nonparametric statistical validation of the results of the 5 phage hosts prediction methods used in this paper. Table S4 lists the average ranks across different taxonomic levels of five methods on all three testing sets, Table S4 presents the average ranks of five methods across various taxonomic levels on all three test sets, with the CHERRY-refined test set results derived from Table 2. The value of $\tau_{F}$ was subsequently computed, as detailed in Table S5, and was found to exceed the critical value of the Friedman test at species and family levels when using a significance level ($\alpha$) of 0.05. However, this threshold was not exceeded at the genus and order levels. The critical values were ascertained based on the number of datasets (three) and the number of methods compared (five), suggesting a significant difference in the accuracy of phage host prediction among the five methods at the species and family levels.

Table S4. Average ranks across different taxonomic levels of the methods on all the testing sets used in this paper.

|  | OUR | CL4PHI | iPHoP | DeepHost | CHERRY |
| --- | --- | --- | --- | --- | --- |
| Order | 1 | 4.333 | 3.333 | 3 | 3.333 |
| Family | 1 | 4.667 | 3.333 | 2.667 | 3.333 |
| Genus | 1 | 4.333 | 3.333 | 3 | 3.333 |
| Species | 1 | 4.333 | 4.333 | 3 | 2.333 |

Table S5. The value of $\tau_{F}$ at different taxonomic levels.

| Level | Order | Family | Genus | Species |
| --- | --- | --- | --- | --- |
| $\tau_{F}$ | 2.991 | 4.924 | 2.991 | 7.963 |

Then the Bonferroni-Dunn post hoc test method was utilized to analyze the differences between the 5 methods quantitatively, specifically at the species and family levels. The value of CD (critical difference) was determined to be 3.224 where the value of $\alpha$ was set to be 0.05. Fig. S3 depicts the chart of the Friedman test of the five phage hosts prediction methods. It is suggested that the proposed method offers a significant improvement over iPHoP and CL4PHI for predicting phage hosts at the species level, while no significant differences were observed between the proposed method and DeepHost and CHERRY at the same level, although the performance of the proposed method is better. Additionally, the proposed method shows a significant improvement over CL4PHI for predicting phage hosts at the family level, with no significant differences observed between the proposed method and iPHoP, DeepHost, and CHERRY at the same level, despite the superior performance of the proposed method.

| 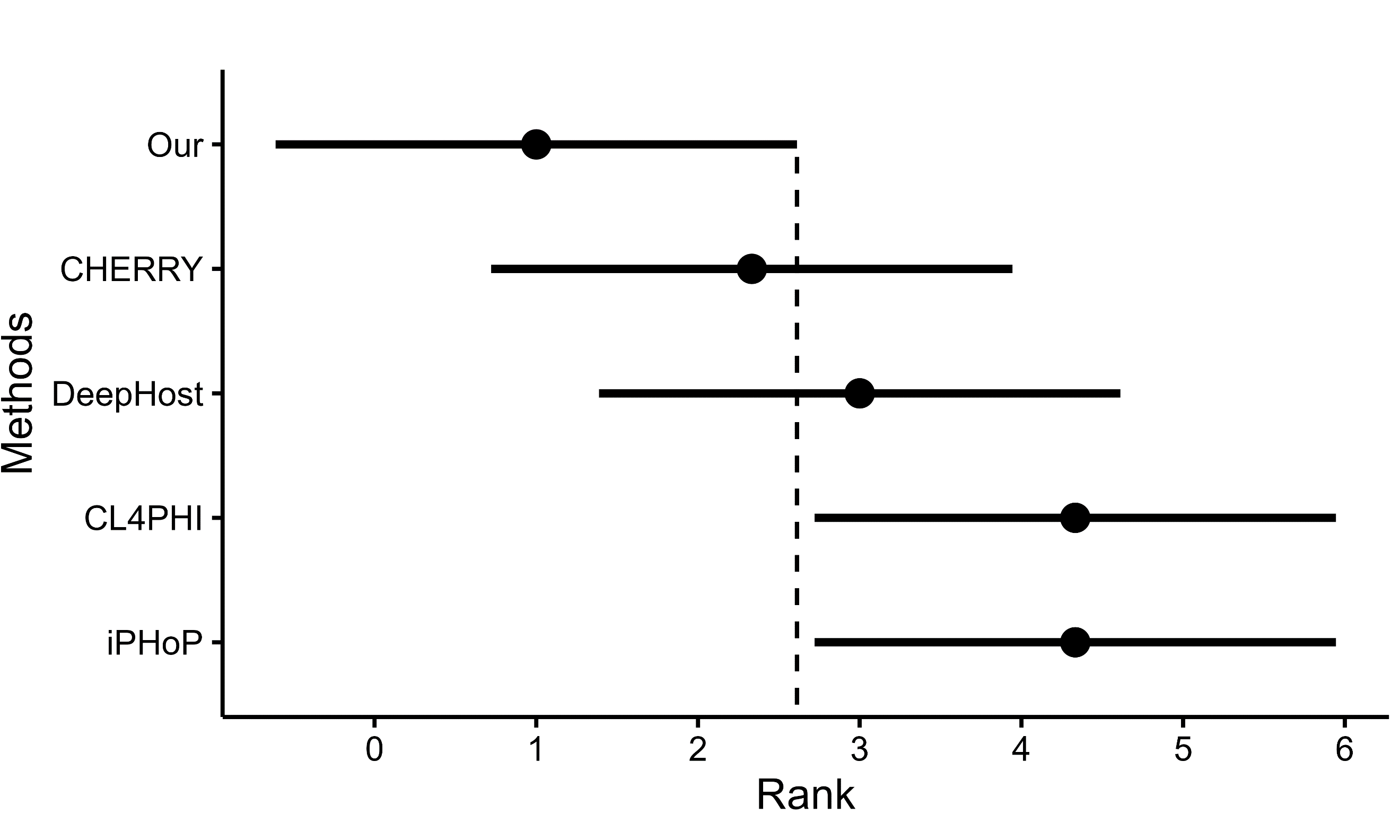  **(A)** | 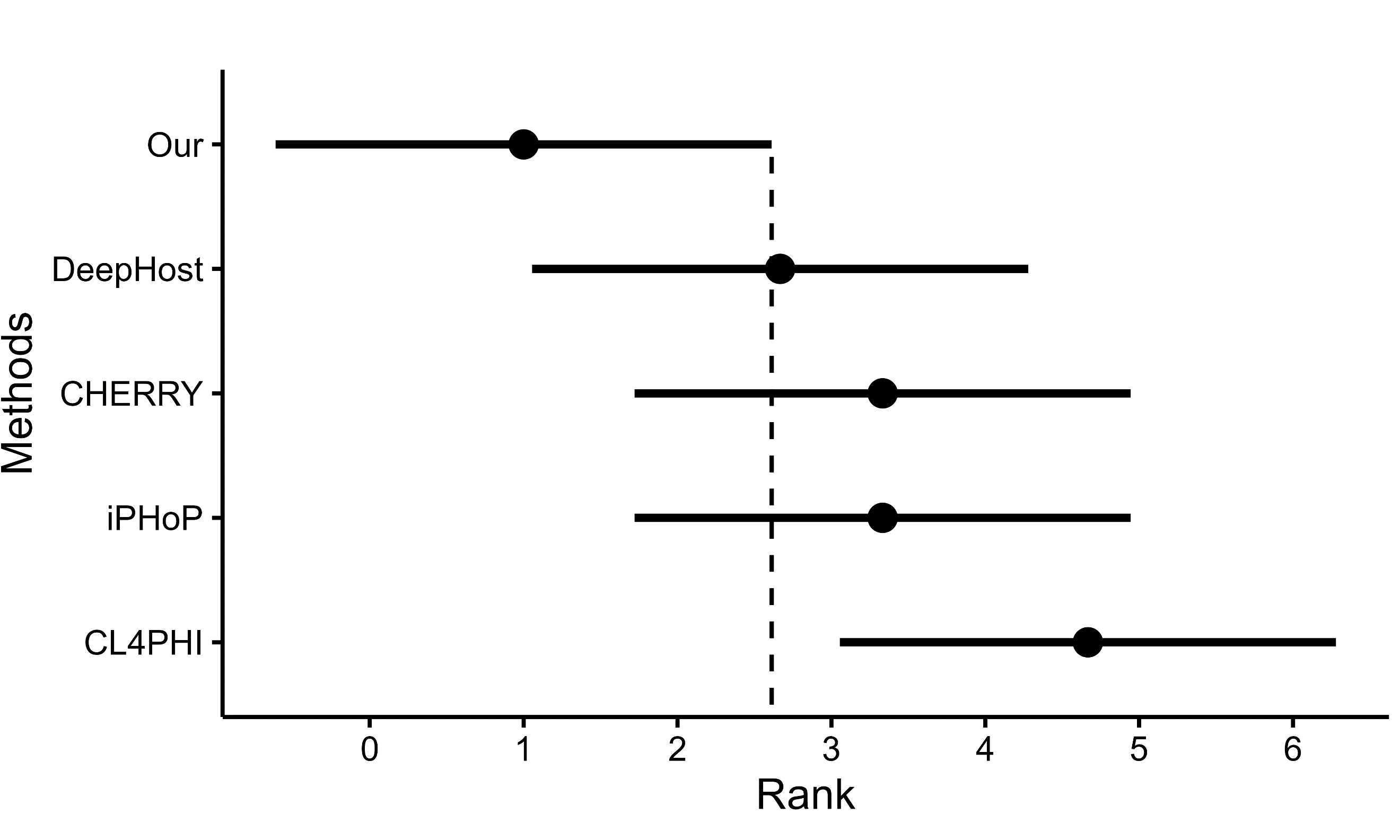  **(B)** |
| --- | --- |

Figure S3. The chart of the Friedman test of the five phage hosts prediction methods at (A) species and (B) family levels. The black dots represent the average ranks of the five methods as listed in Table S4, and the length of each horizontal line is equal to the value of CD.

**Details of model training**

Table S6 shows the default parameters used for training our model. Specifically, Epochs indicates the number of training epochs with early stopping criteria applied when validation performance did not improve over multiple consecutive epochs, thus avoiding overfitting and conserving computational resources, and $M$ denotes the number of attention heads in GATv2. To systematically evaluate the impact of $M$ on the model’s performance, we conducted an accuracy assessment by incrementing $M$ from 1 to 8. As shown in Figure S4, the findings reveal that the model attains its peak performance when $M$ is set to 4. Additionally, to ensure a fair comparison, all other models were trained using the default parameters provided in their respective official documentation.

Table S6. Default parameters for model training.

| Learning rate | Bath size | Epochs | $M$ |
| --- | --- | --- | --- |
| 0.001 | 512 | 2000 | 4 |

**
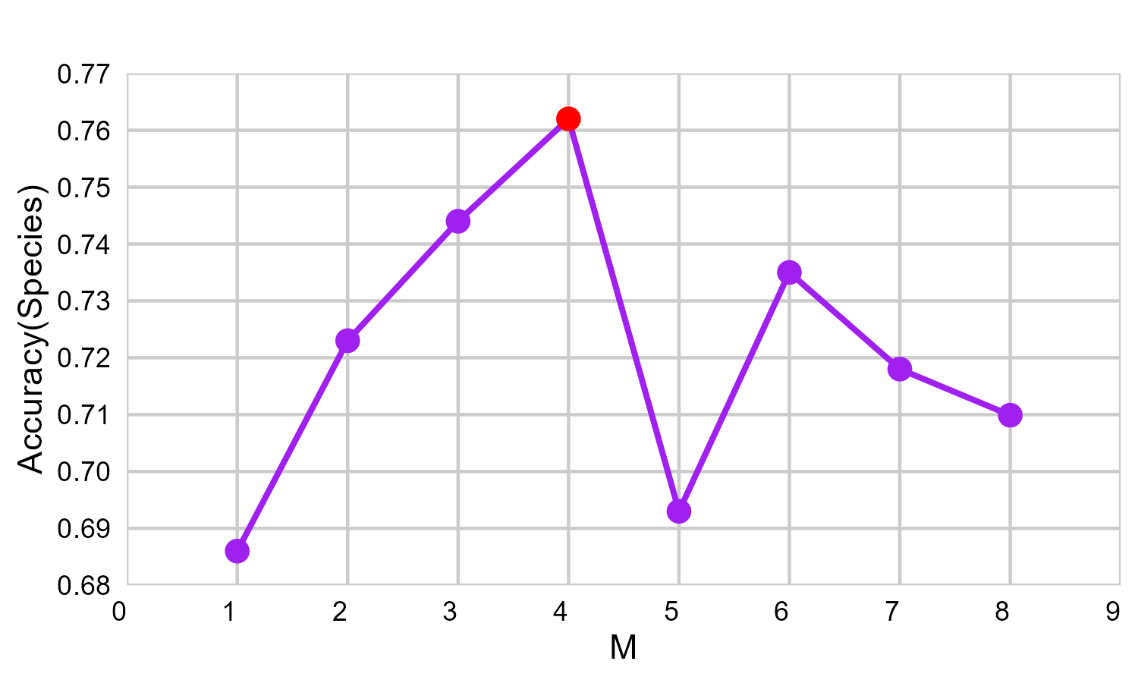
**

**Figure S4.** The effect of hyperparameter $M$ on the host prediction performance.

**References**

1. Sievers, F. and D.G. Higgins, *The Clustal Omega Multiple Alignment Package.* Methods in molecular biology (Clifton, N.J.), 2021. **2231**: p. 3-16.
